# Has anything changed for Fibromyalgia? Focus groups with patients on their lived experiences in England

**DOI:** 10.1371/journal.pone.0342065

**Published:** 2026-02-13

**Authors:** Cosima Locher, Anna Kharko, Joseph W. Lanario, Leo Druart, Kirralise Hansford, Helen Koechlin, Anthony Davies

**Affiliations:** 1 Clinical Psychology and Psychosomatics, Faculty of Psychology, University of Basel, Basel, Switzerland; 2 Faculty of Health, University of Plymouth, Plymouth, United Kingdom; 3 Participatory eHealth and Health Data Research Group, Department of Women’s and Children’s Health, Uppsala University, Uppsala, Sweden; 4 Department of Radiology, Warren Alpert Medical School, Brown University, Providence, Rhode Island, United States of America; 5 Medical Expectations Lab, Brown University, Providence, Rhode Island, United States of America; 6 Department of Women’s and Reproductive Health, Medical Sciences Division, University of Oxford, Oxford, United Kingdom; 7 Department of Psychosomatics and Psychiatry, University Children’s Hospital, University of Zurich, Zurich, Switzerland; 8 Division of Child and Adolescent Health Psychology, Department of Psychology, University of Zurich, Zurich, Switzerland; 9 Children’s Research Centre, University Children’s Hospital Zurich, University of Zurich, Zurich, Switzerland; 10 Pain Management Centre, University Hospitals Plymouth NHS Trust, Plymouth, United Kingdom; Touro University California College of Pharmacy, UNITED STATES OF AMERICA

## Abstract

**Background:**

While awareness of Fibromyalgia (FM) has evolved across healthcare and social settings, individuals with FM continue reporting poor understanding of their condition in the everyday.

**Objective:**

To explore the lived experiences and subjective illness narratives of individuals with FM, focusing on personal understandings of FM, outlook on healthcare, and perceived attitudes of surrounding peers.

**Methods:**

We carried out four online focus groups with individuals diagnosed with FM. Participants were recruited from a pool of patients in a pain management centre in the Southwest of England, UK, that previously took part in a FM management course, ‘Body Reprogramming Course’ (BRC). Discussion topics included the personal understanding of FM (illness identity, timeline, causes, consequences, and controllability), history with FM diagnosis and treatment, attitudes of family and friends towards FM, as well as personal attitude towards medication. Audio recordings from focus groups were transcribed and analysed by two primary coders, using a reflexive thematic analysis approach. Codes were iteratively discussed and refined with two additional coders.

**Results:**

20 individuals with FM took part in four focus groups, aged between 25 and 67 years. Analysis of the data revealed 5 overarching themes and 10-subthemes related to lived experiences of FM: 1) The individual journey; 2) FM in healthcare; 3) FM in social context; 4) FM in personal context; 5) Experiences with BRC. Although participants felt that there has been some positive societal shift towards FM, most discussions concerned personal struggles with conveying to family and healthcare professionals the impact of the condition. Participants described a significant emotional toll of dealing with hidden struggles. Many expressed disappointment with prior care, describing it as fragmented, and some expressed strong opposition to medication-based treatments. Reflecting on the BRC, participants liked the coping strategies and the developed connections.

**Conclusions:**

While participants recognised some positive changes in others’ understanding of FM, the overwhelming sentiment was that FM remains overall poorly received and supported by peers and healthcare professionals. Participants in the BRC described their experience of FM as part of a journey toward a higher purpose, linking their illness to personal growth.

## Introduction

Fibromyalgia (FM) is a debilitating chronic primary pain syndrome characterized by widespread musculoskeletal pain, fatigue, “brain fog” and heightened sensitivity to tactile stimuli, significantly impairing daily functioning and quality of life [1,2]. Living with persistent, diffuse pain is linked to experiencing significant emotional distress and/ or functional disability [2,3] According to the ICD-11 FM is a condition where *pain itself* is the disease [1,4]. The prevalence of FM is approximately 2% globally, affecting individuals across diverse demographics, with a clear predominance among women [5]. This imposes substantial economic burdens on healthcare systems, including the National Health Service (NHS), the publicly funded healthcare system in the United Kingdom [6].

The invisible nature of FM symptoms can lead to feelings of frustration and isolation among those diagnosed patients [7]. Healthcare providers, particularly general practitioners (GPs), may also find managing FM challenging due to its multifaceted presentation and the lack of definitive diagnostic tests [3,8]. This can result in difficult consultations where both patient and physician feel dissatisfied, underscoring the necessity for supportive, open, and reciprocal patient-doctor relationships to improve outcomes [3].

Current treatment modalities yield limited success, with many individuals experiencing minimal relief. Pharmacological interventions often have modest efficacy and are frequently associated with adverse effects, leading to poor long-term adherence [6]. Non-pharmacological approaches, such as exercise and psychological therapies, offer some benefit but have also shown to reveal limited maintaining effects [9].

Notably, the subjective experience of illness is crucial in understanding the lived reality of individuals with FM. It has been shown that personal interpretations of the condition significantly shape their experiences and coping strategies [7].

The bio-psycho-social model, first introduced by Engel [10], provides a valuable framework for understanding the subjective illness experiences of FM patients. Despite being introduced in the 1970s, it remains highly relevant to chronic pain management as it highlights the interplay between biological, psychological, and social factors in the experience of pain [11]. For FM patients, the model underscores the necessity of addressing not just the physical symptoms — such as widespread musculoskeletal pain and fatigue — but also the cognitive, emotional, and social dimensions of the illness. This includes factors such as pain-related thoughts and beliefs, emotional distress, and the impact of social support or isolation. Likewise, cognitive processes can play a crucial role in the development and maintenance of chronic primary pain. Evaluative and interpretive processes — often termed “illness perceptions” — influence how individuals understand and manage their symptoms [12].

The general construct of illness perceptions comprises five core dimensions: beliefs about the identity of the illness (e.g., labeling symptoms as FM), perceived causes, anticipated timeline, consequences, and beliefs about control or cure [13]. These perceptions shape how patients engage with treatment, cope with symptoms, and maintain social and occupational roles. Building on this, Henningsen and colleagues [14] highlight the role of “perception of bodily distress” in understanding chronic primary pain — referring to how individuals interpret and respond to bodily sensations, particularly when these sensations are perceived as threatening, overwhelming, or unexplainable.

Importantly, subjective illness experiences have been shown to correlate with health outcomes. Studies have demonstrated that positive changes in illness perceptions are associated with increased physical activity [15] and reductions in pain and depression [16,17], highlighting the potential of interventions that target these cognitive and behavioural factors to improve both emotional and physical health outcomes**.** Interventions that inform and educate patients as comprehensively as possible can improve understanding, coping strategies, and treatment adherence [18], which, in turn, can lead to more consistent engagement in physical activity, better pain management, and reduced depression. Nonetheless, these findings have yet to be applied specifically to FM. There is still much to learn about how these narratives develop, how they change over time, and how they can be positively influenced to improve care outcomes in FM. Developing treatments that are acceptable to patients by offering a plausible narrative remains an urgent need in FM care. Further research is essential to deepen our understanding of these complex interactions and develop more targeted interventions that address the unique experiences of individuals with FM, including the personal understanding of FM (illness identity, timeline, causes, consequences, and controllability), history with FM diagnosis and treatment, attitudes of family and friends towards FM, as well as personal attitude towards medication..

### Objectives

The study primarily aimed to explore the subjective illness experiences of individuals with FM. For this aim, we included patients who took part in a multimodal management for FM, called ‘Body Reprogramming Course’ (BRC) [19]. We decided to focus on BRC because, in this course, patients were actively involved in the development of the FM narrative that is provided to the patients (for details see [19]).

Reflecting the recruitment strategy, as a secondary aim we carried out a focused analysis of instances where individual illness narratives were connected to the BRC.

## Materials and methods

To address the aims, from the 2nd of February to the 3rd of March 2021 we carried out four online focus groups with adult FM patients who previously attended the Body Reprogramming Course (BRC) in the Plymouth Pain Management Centre, Plymouth, UK (hereafter referred to as the Centre). The study is reported following the Standards for Reporting Qualitative Research (SRQR) Checklist [20].

### Qualitative approach

We adopted a thematic analysis approach [21,22] focusing on better understanding the lived experiences of people with FM and their perspective on FM as an illness. We designed the focus group schedules to better understand how contextual factors (e.g., previous healthcare experience, social surroundings, illness perception), shape individuals with FM experiences.

### Context

At the time of the study, there were approximately 2.5 million people in the UK diagnosed with FM, depending on diagnostic criteria used [23,24]. The study was carried out in the South West of England. The region is largely rural, with several cities, of which the second largest is Plymouth. The University Hospitals Plymouth NHS Trust, where the Centre is located, provides secondary care healthcare services to nearly half a million people from both urban and rural backgrounds. The pain management centre is an interdisciplinary clinic providing support for chronic and cancer pain management. Among the services offered in the Centre is the BRC. The BRC is a clinically led pain management course with the aim of helping patients to understand FM and their symptoms using the Hyland Model, which has its scientific origin based on Adaptive Network Theory [25]. The Hyland Model suggests that biological and psychological factors form part of the same complex adaptive control system. The model uses the concept of ‘stop signals’ to help explain FM symptomatology. The course uses this conceptual framework to guide patient related learning about the body and pain, helps develop skills to teach the body that movement is safe, as well as techniques around learning how to handle difficult thoughts and feelings and painful experiences more effectively. The BRC incorporates lifestyle and values-based management strategies as well as sessions that teach patients on how to cope with symptoms in a compassionate way. Finally, the program introduces relaxation and body-based techniques – including gentle movement practices such as Tai Chi and Yoga – to help calm the body and re-establish a sense of trust in its capacities.

### Participant recruitment

Potential participants were recruited through the Centre. Eligible participants were individuals diagnosed with FM who: 1) were 18 years and older; 2) had visited the Centre in the last 12 months; 3) had attended the BRC in the last 12 months; and 4) were able to participate in the focus groups online.

A list of eligible participants was compiled by AD and two staff members of the Centre, who were not part of the research team. Recruitment was guided by convenience and purposeful variation sampling strategy with the goal of inviting participants of various age, gender, and BRC attendance modes (i.e., online or in-person). Participants were informed of the forthcoming study at the end of their course and those who expressed interest were sent the study information sheet and consent form. Study invitation packages were sent via post by the Centre staff members, and included information about the study and a consent form with a prepaid return envelope. Once a completed consent form was returned, Centre staff members forwarded the participant contact details to the research team. AD, CL, and AK then invited the potential participant to choose a focus group time slot. Potential participants who did not complete a study invitation package were not contacted further. Those participants who consented but did not select a time slot, as well as those who selected a time slot but did not show up, were sent a repeat email invitation to pick a time slot.

The recruitment period started on the 2^nd^ of February 2021 and ended on the 25^th^ of March 2021. All participants provided written informed consent and were able to withdraw from the study at any point without penalty. Participants were not compensated for participation.

### Participant characteristics

As a result of the recruitment efforts, four focus groups with a total of 20 participants were carried out (see Table 1). Focus groups varied in size, with a minimum of 3 and a maximum of 7. Most participants were women, aged between 25 and 67, with a mean age of 46.1. Participants had been diagnosed between 1 and 25 years ago. Of these, 14 individuals (73.6%) had received the diagnosis two years ago or less, and 5 individuals (26.3%) more than two years ago, with a median time since diagnosis — 2 years. In total, 80% of the participants had attended the BRC online (see Table 1).

**Table 1:** Participant characteristics (*n* = 20).

Focus Group	Participant	Gender	Age	Years since FM/CSS diagnosis	BRC Attendance
1	1	Woman	45	25	Online
1	2	Man	48	3	Online
1	3	Woman	49	2	Online
1	4	Woman	46	1	Online
1	5	Woman	42	4	Online
1	6	Woman	40	7	Online
1	7	Woman	25	2	Online
2	8	Woman	48	2	In person
2	9	Woman	59	18	In person
2	10	Woman	38	2	In person
2	11	Woman	43	2	In person
3	12	Woman	54	6	Online
3	13	Woman	50	2	Online
3	14	Woman	49	2	Online
4	15	Woman	49	2	Online
4	16	Woman	67	1	Online
4	17	Woman	32	1	Online
4	18	Woman	55	1	Online
4	19	Woman	41	2	Online
4	20	Woman	42	2	Online

### Data collection

The focus groups were conducted online via Zoom, as video meetings. Group facilitators were CL and AK, and were assisted by Beth Allen, a Bachelor student who provided technical support. Upon joining the online meeting, the details from the study information sheet were reiterated, and it was confirmed that each participant provided informed consent. A single-slide presentation detailing the rules for the focus group was then shared (see Appendix, S1 File). Once the recording began, CL and AK took turns in asking the group questions following the interview guide (see **Box 1)**.

**Box 1. Interview guide.**

1 What does FMS mean to you?

**Probe:** How do you explain to friends and family your illness? Has your understanding changed since you attended the ‘body reprogramming’ course? [illness identity]

2 What is your explanation why you are ill?

**Probe:** Do you have any ideas how it started (e.g., triggers)? Has your understanding changed since you attended the ‘body reprogramming’ course? [causes of the illness]

3 How has the way you have managed your illness changed from before to after attending the ‘body reprogramming’ course? [control of the illness]
4 How do you see your future?

**Probe:** What would that look like for you? Has this changed before to after attending the ‘body reprogramming’ course? [outlook]

Each focus group was between 80 and 90 minutes long, with a 10-minute break after 60 minutes. Owing to the nature of FM, participants were encouraged to move or take breaks as necessary. Once the focus group discussion concluded, the recording was stopped. The video portion of the recording was deleted immediately, leaving only the audio recording for further processing.

### Data processing and analysis

The audio recordings were transcribed verbatim by KH, with the transcription being performed manually without the use of any specialized tools or applications. Transcriptions were then uploaded for analysis to QCAmap, a web-based software designed for qualitative coding and analysis. This tool was used to organize the coded data, facilitate collaborative coding efforts, and track the development of categories and themes throughout the analysis process. Data were analysed following a reflexive thematic analysis approach [22], which involves a flexible, iterative process that emphasizes researcher reflexivity and interpretation. Researchers maintained an active, reflective role in shaping the themes and categories rather than simply applying pre-existing codes [22]. Due to the chosen analysis approach, data saturation was not applied, as it is not considered appropriate [26]. Instead, sample size was guided by the concept of information power [27]. Taking into account the specificity of the sample (participants who previously attended BRC), the study objectives (subjective experience with FM and BRC), and the quality and richness of the focus group discussions (observed repetition of narratives in the focus groups, FG, 3 and 4), we deemed data from the four focus groups to be sufficient for analysis. KH and LD were primary coders, assisted by CL and AK. Analysis started with KH and LD assigning low-level codes to short passages of text. These were then discussed in an iterative manner with CL and AK with the aim to refine the codes and create higher order categories. The initial codes created by KH and LD were updated and adapted by CL and AK through joint discussions. Coding disagreements were handled within coding pairs by consulting the original transcripts and reflecting on the conversational contexts. Where it was not possible to reach a consensus, discussions were held with the wider research team. This process prevented the formal calculation of inter-rater reliability. Once the categories were finalised, they were placed under preliminary themes. Following further iterative discussions, the themes were finalised.

### Researcher characteristics and reflexivity

The research conduct and analysis were likely influenced by the team’s pre-existing beliefs about FM and more widely chronic pain management. The authors considered how their previous experiences offered insight and potentially introduced bias. CL is a female healthcare researcher who was funded by the Swiss National Science Foundation to conduct her research abroad at the time of the study. CL’s doctoral and postdoctoral research focused on Chronic Primary Pain. CL is trained as a psychological coach. AK is a female healthcare researcher, who was completing her PhD in Psychology at the time of the study. AK’s doctoral research focused on FM, and she had second-hand personal experience with healthcare services for FM in South West England. LD is a male physiotherapist with some clinical experience in managing FM. His research involves chronic pain management and employs mixed-method approaches. KH is a female chronic pain researcher, who at the time was completing her postgraduate degree in neuroimaging. CL and AK were involved in study design and analysis, while KH and LD were involved in analysis only. JL was involved in study design and has published previously on the use of BRCs for FM and asthma, but was not involved in the analysis described here. AD is a pain medicine specialist and previous lead clinician within the Centre. He has a subspecialty interest in Fibromyalgia and other Central sensitivity syndromes. Overall, these backgrounds contributed to sensitivity in interpreting participants’ accounts, particularly in relation to clinical practices and patient-clinician interactions. At the same time, they may have introduced assumptions about FM management, which the team sought to balance through reflexive discussion and consideration of alternative interpretations.

### Ethical considerations and data management

The study received ethical approval from Health Research Authority (HRA), London-Riverside Research Ethics Committee (REC Reference # 19/LO/1296, IRAS ID: 266140) prior to any recruitment and data collection. The study was carried out in accordance with the Declaration of Helsinki. With regard to inclusivity in global research, see Appendix (S2 File) for details. Gathered data consists of anonymised transcripts, and demographic information from clinical records. Due to the potential for participant deanonymisation, data are not available for sharing.

## Results

Our thematic analysis resulted in 10 sub-themes grouped into 5 major themes as shown in Table 2.

**Table 2:** Themes and sub-themes.

Theme	Sub-theme	Description
1. The individual journey	Constant challenges	Participants described a lifelong series of hurdles, which were driven by the condition’s unpredictable course, eroding sense of independence and fuelling worries.
	Severe emotional toll	FM was experienced as emotionally devastating, bringing sadness, anger, vulnerability, and helplessness.
2. FM in healthcare	Fragmented care	Care was perceived as siloed, with symptoms often dismissed or attributed to FM. Trust in clinicians was fragile, though a few validating, investigative providers were also present.
	Medication aversion	Wariness towards medication was present due to limited benefits, side effects, and fear of dependence or altered sense of self. Some refused medication or felt trapped by withdrawals and reliance.
3. FM in a social context	Hidden struggles	FM was seen as invisible, which led to feelings of isolation. To avoid potential stigmatisation or burdening others, participants often concealed the severity of their symptoms.
	Finding understanding	Social experiences ranged from loss of relationships to gain of support networks. When others expressed understanding towards FM, participants felt freer to be honest and set expectations.
4. FM in a personal context	Strength in purpose	Participants sought meaning in their experience, sometimes linking FM to years of self-sacrifice and overexertion.
	Coping strategies	Coping strategies were common and centred around self-acceptance, pacing and rest, and when possible gradual movement/exercise.
5. Experiences with BRC	Course content	BRC was seen as valuable for providing explanations and helpful metaphors. Potential improvements included more medication discussions and personalisation to attendees needs.
	Creating connections	Group sessions were a highly valued aspect of BRC as they offered validation and peer learning, though were sometimes overwhelming.

### Theme 1. The Individual Journey

#### Sub-theme: Constant challenges.

We began the focus groups, asking the participants to reflect on their history with FM so far. Overwhelmingly, participants described their past as a series of ongoing challenges in the fight for a better quality of life. This would begin early in the diagnostic journey with the symptoms being initially attributed to a variety of conditions other than FM:

*- “was always put down to, oh growth pain, oh you will grow out of it, nothing about it”* (Focus Group 1, FG1)

*- “kept being told it was chronic back pain, arthritis, this that and the other”* (FG2).

FM symptoms would commonly be attributed to personal shortcomings rather than a medical condition. One person remembered: “*when you’re a teenager, [you] just get told you’re lazy”* and later in life when attempting to work *“you don’t want to go to work, and you haven’t got no work ethics, and it’s like no, I just physically can’t do it, my body is screaming in pain*” (FG3). Some had to give up working due to FM (*“had to give up work completely in July 2019 because my fibromyalgia was really affecting, physically affecting, how I was working*”, FG3). Those with families felt they couldn’t carry out their parental responsibilities, which made them “feel guilty”:

*-* “*because my sons have had to do an awful lot more than most children. You know, trying to get mummy out of bed so she can take them to school when they are seven years old is not nice for any child*” (FG1).

Others gave up on leisurely activities that brought them meaning, e.g., one person said they “*used to be quite sporty. And now, I can walk a little bit, I can do a little bit of cycling. It’s not satisfying, frankly*” (FG4). Failing to fully engage with life responsibilities and pleasures was linked to loss of identity: “*for me, it was a loss of independence, and a loss of me as a person that I was*” (FG2).

Added to the challenge was that FM was seen as ever-changing. For some, it was *“like a rollercoaster”* (FG3) with good and bad days, which inspired the feeling they were “*still on a quest to get better*” (FG1). Living with FM felt like *“such a difficult journey to understand our bodies and what works for us and what doesn’t work for us”* (FG2). And that would not “*happen overnight, it is small steps.*” (FG4) For others, the dynamic brought up by FM only added to the burden: “*it is just the unpredictability of fibro which is difficult”* (FG3). For many their well-being has been on a steady decline despite their efforts (*“slowly getting worse again”*, FG1). This uncertainty in FM progression left individuals apprehensive about their future:

*-* “*Worried about what the outcome is going to be in like 5, 6, 7 years. I’ve got a 16-year-old daughter at home, and I’m worried about if I am going to be able to play with my grandchildren.*” (FG2)

#### Sub-theme: Severe emotional toll.

As participants recounted significant life developments, it became evident the profound impact FM has had on them emotionally. Living with FM was described as something that “*emotionally destroys you*” (FG1) and “*a very depressing, soul-destroying feeling*” (FG1). As participants took turns to share their stories, one said that the name alone was upsetting enough: “*the word fibromyalgia brings the tears, and then you get angry*” (FG1). Adding to this was the sense of vulnerability that FM creates “*because you’re at the mercy of everybody because you want to be helped, you want to understand*” (FG2). Despite wanting to get better, some felt helpless:

*-* “*I see no point contacting anybody anymore. My husband says you should be in hospital right now, what’s the point, what are they going to do? There’s nothing anybody can do, so why fight it?*” (FG1).

Participants gave several examples of how FM had a widespread impact on their life and forced recurring major life adaptations from them:

*-* “*I had to give up work completely in July 2019 because umm my fibromyalgia was really affecting, physically affecting, how I was working*.” (FG3)

*-* “*I have to take voluntary redundancy because of fibromyalgia because I end up getting chronic migraines with it*” (FG1)

*-* “*I feel guilty because my sons have had to do an awful lot more than most children, umm, you know, trying to get mummy out of bed so she can take them to school when they are seven years old is not nice for any child.*” (FG1)

### Theme 2. FM in Healthcare

#### Sub-theme: Fragmented care.

Perspectives on how FM shaped experiences with healthcare was a focal discussion topic. As a whole, participants described the healthcare system as lacking a cohesive holistic approach when it comes to treating FM. As patients, participants felt that healthcare professionals often failed to consider all of their symptoms:

*-* “*most of the time they don’t look at you holistically*” (FG2)

*- “they just look at one thing, and it’s an absolute nightmare that they can’t look at you as a whole person”* (FG2).

When particular symptoms were discussed with a clinician, individuals felt little support was offered to them (“*they couldn’t pinpoint anything so they just said “You’re just going to have to live with it, deal with it*”, FG1). It seemed for many that FM became the go-to explanation for any health complaint:

*- “Every time you have something wrong with you, and you go to the doctors, they just go fibro. Fibro. And it is like, I could be dying, but you’re not actually checking!*” (FG4).

Met with such attitudes, individuals with FM felt abandoned by the healthcare system (“*a very lonely thing*“, FG1).

In contrast, some faced distrust from their healthcare providers. One person shared that they experienced that already in their adolescence, doctors would dismiss their health concerns:

*- “When I was in my teens, I went to a GP and said what’s wrong. She said, “Oh, there is nothing wrong with you, just get a life. There is nothing wrong with you get on with it. But I know something, it is not normal to feel so sleepy all of the time.*” (FG4)

Participants reported that their lived knowledge about FM was often dismissed, feeling that “*[doctors] don’t like it when you’re a bit more of an expert patient than they are. I have an awful lot of problems with that.*” (FG1) Unsurprisingly, a distrust towards healthcare professionals themselves was recurrently mentioned. Participants felt there were only“*a few great doctors that completely understand what you’re going through*” (FG2). For most participants, their experience with healthcare professionals left them largely disappointed. This ranged from doctors being *“a bit dismissive, not in a rude way*”(FG3) to doctors that have made participants *“feel less human*” (FG2), some being told that *“fibromyalgia is a make-belief illness and that it’s not real*” (FG4). The reason for the poor attitudes from doctors was unclear, but some individuals believed it could be because “*they didn’t have the time or didn’t have the knowledge*” (FG3). Due to this, adhering to proposed treatment plans was challenging: “*[doctors] will often make a decision and expect you to do something, and I don’t know if what they’re asking is going to make me worse?*” (FG2) Still, participants acknowledged there were specialists that were able to offer support for FM (“*fantastic doctors [...] will do anything that they can for you. And they’re the ones that make you feel like it’s real*”, FG2). As patients, these were the type of doctors needed for FM management:

*- “They do their best to help you. They don’t cluster everything as fibromyalgia. You go to them with symptoms, and they don’t automatically say, oh it’s your fibro. They will investigate it*” (FG2).

Notably, individuals with FM appreciated that they had different and positive experiences with healthcare professionals in the BRC program, for example mentioning that it “*makes all the difference*” because “*suddenly you were hearing someone who understood what he was talking about*” (FG2). This is explored further in theme 5.

#### Sub-theme: Medication aversion.

When it came to managing FM through medication, many participants expressed scepticism and distrust. While many had a long history with a variety of medications, participants felt uncomfortable taking them now and actively avoided them: *“I’ve been there a couple of times but due to my experience I’d rather cry myself to sleep than ever take medication again”* (FG1) and *“I try and do everything I possibly can to avoid taking the strong medication because it does frighten me”* (FG2). This was closely linked to the worry of becoming dependent or even addicted (*“absolutely terrified of becoming an addict”*, FG4). Those who had more experience with medication felt it changed them as a person (*“Because of medication, I’m not me anymore”*, FG1). The experience with a variety of prescribed drugs had such a negative impact that some participants outright refused taking any: “*you can not tell me, or force me, or no matter what you do, how much you explain it to me, I will not take it. I refuse to take it. Because I know what it leads to*” (FG1). In one case, this rejection extended beyond the person themselves and to their family as well:

*- “I’m actually just trying to teach my son at the moment because he has just done his knee in, umm, and he wanted me to get him sleeping tablets and I refused. Umm, I said no I’m sorry darling but whether it’s a torn ligament or whatever you’ve just got to keep persevering with it.”* (FG1)

Participants linked this aversion to the extensive list of side effects they had experienced. For example, one individual remembers how a prescribed drug “*knocked me out and in the morning I could not wake up, I spent the entire day just drifting in and out of sleep.*” (FG1) Another also described how another prescription would “*put me in an altered state. They help the pain, but they just put me in an altered state, so that’s not a solution*” (FG4). For many, “*the side effects way outweigh the benefits*” (FG1). Some participants described feeling unable to discontinue medication:

*- “you have horrific side effects when you stop taking them. Like the withdrawals from them, they are not nice at all*” (FG2)

*- “I now rely on that medication so much that to stop it would actually be worse on my mental health. It would be worse on my physical health. So I’m now stuck in this*” (FG1).

Participants expressed that to them; the limited benefits coupled with the experienced or potential side effects left them wondering what else is available for FM symptom relief (*“I’m worried I’m going to get to the stage where there’s nothing left for me to take*”, FG2; “*frightened of being without*”, FG3).

### Theme 3. FM in Social Context

#### Sub-theme: Hidden struggles.

As most of FM management is self-management outside the healthcare context, participants’ experiences with FM in society as a whole were also considered. A central discussion point was the invisibility of the condition and the hardships that arise from it:

*-* “*when you haven’t got something that people can physically see, they don’t understand it”*

*-* “*they think you’re putting it on*” (FG1).

Failure from those around to appreciate the challenges that FM creates, had led to many feeling lonely and misunderstood:

*- “It’s difficult to explain. It is not just fatigue, it is really bad fatigue*” (FG3)

*- “You feel you have to keep going because no one is asking you if you’re okay*” (FG2).

When discussing, how could an individual with FM explain their condition to those around them, an objection was voiced to doing so:

*- “[Don’t want to let] them know how bad I am because I don’t want them to worry*” (FG2),

*-* “*I don’t want to come across as if I’m whinging, hypochondriac*” (FG2).

The worry of losing the social circle was a prominent reason for keeping to oneself: “*really I just want to go, I’m feeling so shit! But you can’t do that to people, otherwise they will run a mile*” (FG3). Similarly, the fear of feeling disappointed by their social circle’s response was also discouraging (*“because it can be quite upsetting when you don’t get the response you need*” (FG2).

#### Sub-theme: Finding understanding.

When describing their social life, participants had a variety of experiences. Some felt they lost their social connections due to FM (*“I have no friends, nothing because it is like fibro walked in and everybody has just walked away*”, FG4). Still, those who had retained the connections, would also feel lost:

*-* “*They can’t understand they’re like if you could do it yesterday why can’t you do it today? And it’s trying to get them to understand how you can be completely different in a few hours or in 12 hours’ time you can be a completely different person” (FG3).*

The doubts of the social circle would sometimes be reflected in the person with FM. One participant confided, they thought to themselves *“you sound like you’re making this up, this doesn’t sound like it can be real for anybody”* (FG2). Such self-doubts could last “*for a very long time [when] you believe what other people are saying or hinting that, that it is your imagination, or you’re lazy or something that is your fault. You know, you are not eating properly [...], or you’re not drinking enough or whatever.*” (FG3) In contrast, other participants shared that they had a strong support network despite their FM. Their family and friends managed to develop an understanding of FM: *“I’ve asked them to Google, to research it a little bit, because it was easier than me trying to explain, and they’re very good, they know if I can’t make something, they’re fine. They understand.*” (FG2) Thanks to the shared understanding, these individuals felt empowered to be honest with their social circle, for example:

*- “I am able to say to them ‘Oh, I am having a foggy day, can’t speak at the moment. Foggy day, I will give you a ring in a couple of days’, something like that”* (FG3).

### Theme 4. FM in a personal context

#### Sub-theme: Strength in purpose.

A major theme was also the participant’s own relationship with FM. In the process of describing their feelings towards the diagnosis and all that it brings with it, the need for a bigger meaning was often brought up. One person described it like that: *“I’m a big believer that God made me with big shoulders and big hips for a reason, as my grandma would say*” (FG1). Many agreed on the possibility that they may have developed FM due to a long history of sacrificing their own needs over others’ (“*... looking after everyone else. Now my body is giving up on me. And that’s why I’ve got fibromyalgia, or I’ve always had it probably, but I have always had to put it to the back*”, FG1). One person even felt they could recall the point it happened (*“to the point I kept pushing myself and pushing myself, to having a breakdown*”, FG4).

Despite some participants mentioning they believed that FM developed as a result of self-exertion, many continued to push themselves. One person described:

*-* “*When I’m having a really good day, I try and do as much as I can in that day. Of course, then I suffer from it a few days afterwards because I have done too much*”, but in turn “*when I am having a good day, I want to cram everything in because I am afraid, I am going to miss out for the next 4 days”* (FG3).

#### Sub-theme: Coping strategies.

In order to persevere, participants described they had developed their own coping strategies. (“*learned to deal with my condition in my own way*”, FG1). Some found it was crucial to accept the limitations brought up by FM:

*“living in the now actually helps. I’m hoping that, if things don’t get better, at least they won’t get worse. I know that it’s a full-time job really, looking after yourself, with this condition*” (FG2).

This could include listening to their own body *“If I am having a bad day, to say I am having a bad day and that is it*” (FG3). Others described slowing down and learning to rely on others where possible. Pacing was seen as challenging but necessary, e.g., *“when it gets really bad [I] try to think: you know I’ve been here before, resting does work. And it does mean that things will change*” (FG2). Opposite to that, others found that increasing exercise was beneficial to“*building up gradually my movement, my exercise and I think that helps*” (FG2). Beside movement, gaining knowledge about FM was seen as important in managing it. For example, *“understanding its scientific base for me … it just removed all of the guilt and self-blame”* (FG3). Even though learning about FM does not relieve it, it was still valuable to participants (*“the pain doesn’t necessarily lessen, I just understand it more and can cope with it better”,* FG3).

### Theme 5. The Body Reprogramming Course

#### Sub-theme: Course content.

Lastly, participants recalled their experiences with the BRC and how that has influenced their perspective on FM. Participants enjoyed the content, describing it as “*very-very helpful*” (FG1) and informative (“*to me, information is so key*”, FG1), and allowing them to develop *“a newer understanding of the condition*” (FG1). The change in mindset was described as particularly beneficial: “*the whole concept of the course really was to make us understand in our minds there was a different way of thinking about what was happening with us*” (FG1). As the course was delivered by a healthcare specialist, having them throughout the course was viewed particularly positively: “*just to know that we have someone like him on our side, that’s a big bonus*” (FG1).

The metaphors offered in the course were adopted by some to help them bridge the communication gap between them and their social circle. Talking about the software-hardware metaphor, one person described how it could help: *“if I was explaining it to anybody else, my granddaughter for example, that is the analogy I would use. Because it is very descriptive, it is a very good way of explaining what is going on in the body.”* (FG3). The mental exercises were favoured by some for their simplicity, e.g., “*listening and closing my eyes for five minutes and blocking the kids out, yeah that works*” (FG1). Importantly, not all techniques worked universally for everyone. For example, picturing oneself as a leaf or twig floating down a water stream *“was given to us as a tool to help us to relax our bodies and our minds together. […] I do it every night before I go to sleep, and I found it a method of being able to go to sleep much better and much quicker”* (FG1). For others, it did not work, one person saying “*trying to pretend I’m a twig and going down a river really doesn’t work for me”* (FG1).

Further to this, participants saw opportunities, in which the course could be improved. For example, the way in which medication was approached: “*the way we were looking at the drugs felt really overwhelming to me. When you have people calling out answers, and they are all put up on the board”* (FG2). Introducing other components to the course was also suggested. This included carrying out counselling in parallel with the course (*“linking counselling sessions along with the course, I think would probably benefit a lot of people”*, FG1). In terms of organisation, participants had the input that supplementary materials would be beneficial (*“there were no handouts and there was no there were no supporting information*”, FG2). Participants particularly enjoyed the social aspect of the BRC and would have preferred to have more time dedicated to it:

*- “During the break I needed to rest, I needed to not talk to anyone, but sometimes it was the only time you got to talk to the other people. It always felt as if I can learn a lot from these people because they are experiencing what I am”* (FG2).

It was also proposed that the BRC was partially carried out in a personalised, individual manner. Building on that, it would have been beneficial for individual feedback “*come up with an action plan to help them utilise the [BRC] tools*” (FG1). Participants also felt people with FM should be included in the future iterations of the course, e.g., *“at the stage of when you’re planning, when you’re about to write it all, adapt it, having that voice of the person with the condition in that room with you in some form is invaluable*” (FG2).

#### Sub-theme: Creating connections.

Beyond the content of BRC, one salient opinion was regarding the social aspect of the group-based sessions. Because participants were grouped with other FM-diagnosed peers, they felt it *“helped me join a group of people that I can now vent to when I’m upset and angry that will fully understand”* (FG1). This was even stated as the most important part for some participants who believed that they “*already knew everything, but what really changed for me, the BRC specifically, was seeing other people. Like [Participant] said, you’re not alone”* (FG1). For some participants, “the main thing that helped me and with the mentality is to learn from other people s experience.” (FG1) This created a sense of community that persisted beyond the course: “*we had a WhatsApp group that went on for quite a while”* (FG2). Some group members even formed long-lasting friendships (“*we have knitted quite a close friendship with the rest of the guys, you know, and that is very helpful for us”*, FG4). However, at times, the group setting was also tiring. For instance, when “*people were talking about what they were on, and it was just, I found it really overwhelming”* (FG2).

## Discussion

The present study explored the subjective illness experiences of individuals with fibromyalgia (FM) who participated in a multimodal intervention called the ‘Body Reprogramming Course’ (BRC). The findings revealed profound insights into individuals with FM lived experiences, which were categorized into five overarching themes, namely the individual journey, FM in healthcare, FM in a social context, FM in a personal context, and the BRC.

Despite advances in perception of FM in social and healthcare settings, participants described persisting lack of in-depth understanding of both the condition and its impact on individuals. Living with FM was seen as a continuous struggle, marked by diagnostic delays, misunderstanding of symptoms, profoundly impacting on daily life. The unpredictable pattern of FM contributed to a sense of loss, as it prevented from engaging in daily obligations and leisurely activities. In the healthcare setting, participants described their previous experience with care as fragmented and poor. Individuals with FM felt that due to their condition, they continued being dismissed, with many describing to have lost trust in healthcare professionals. Importantly, some had positive experiences with healthcare too, although overall discussions centred around a sense of helplessness.

It is notable that opposition to medication was repeatedly brought up in the groups. This is in line with other research that suggests individuals with FM have limited expectancies about pain relief from pharmacological treatments [28]. Pain relief medication was seen as offering little reprise in comparison to the potential dependency and side effects. This aversion contrasts with long-standing clinical assumptions that individuals with chronic pain actively seek pharmacological treatment, even when it carries risks of long-term dependence, as with opioids [29]. The shift in our participants’ opinion may reflect rising awareness within the local FM community of the limited benefits of long-term pharmacological pain management. Beyond healthcare, discussions centred around the seeming societal invisibility of FM. Misunderstandings were common from family and friends, which made some participants socially withdrawn and reluctant to share their struggles. In contrast, others have been able to form support networks that fostered understanding and acceptance, particularly once they managed to educate their family about FM. Despite the prevalent negative views of FM, some individuals also demonstrated an illness narrative grounded in resilience. Many participants shared various coping strategies, such as pacing and mindfulness-based techniques, to cope with FM. Some participants also expressed the need to find a higher purpose in their experience with FM. This meaning-making process appeared to serve as a psychological resource, allowing individuals to reframe their suffering. The finding is in line with established meaning-making frameworks in chronic illness, which describe how individuals re-evaluate life goals, reconstruct identity, and reinterpret suffering to preserve coherence and agency despite ongoing symptoms [30].

The sub-themes can be interpreted through the lens of the questions asked during the interviews, which were aligned with the five core dimensions of illness narrative [23]. *Beliefs about the identity of the illness* were affected by the delayed diagnosis of FM and the constant misattribution of symptoms. Some participants were concerned that FM had become a “catch-all” diagnosis, with healthcare professionals dismissing any new health complaints as simply part of FM. *Perceived causes of illness* were discussed when considering personal origin of FM, with many believing that it may be due to long-term overexertion. The idea that FM was developed as a consequence of continuously prioritising others was common. *Anticipated timeline of the illness* was captured through many participants’ concerns over the unpredictable progression of FM (*“like a rollercoaster”*). The lack of clear prognosis created feelings of apprehension about the future, with some fearing ultimately a significant deterioration. *Consequences of the illness* were the most discussed dimension, with all areas of life seen as being impacted. Feelings of guilt, fear of missing out on meaningful life activities, withdrawal, and loneliness were voiced throughout the focus group sessions. Perhaps most striking was some individuals’ sense of loss of their self-identity as a direct result of FM. Lastly, *beliefs about control or cure* could be seen to have emerged in scepticism towards healthcare and aversion towards medication.

Nonetheless, participants shared positive perspectives as well. When asked about their experiences with BRC, the course was largely viewed as beneficial. It was seen as helpful in two main avenues. First, it helped participants to reconceptualise their condition and equipped them with practical coping strategies, as well as provided useful metaphors which aided explaining FM to their peers. Above all, participants valued the group setting of the course, which allowed them to form connections with other diagnosed people with FM. Thus, it helped individuals to realise that they are not alone with FM. Some suggestions were offered to make the course less intensive and more individualised, for example the inclusion of patient representatives in the process of developing intervention content, something that is increasingly asked for in pain research.

Our findings align with previous research on experiences of individuals with FM, emphasising the complex emotional burden, social isolation, and fragmented care [11,31,32]. Comparable to previous literature [33], participants in the present study reported a pervasive sense of abandonment within the healthcare system. The positive impact of peer support and self-management strategies aligns with evidence indicating that social connections and self-efficacy play crucial roles in managing FM [34]. Likewise, meta-analyses reveal that multi-component treatments are effective for individuals with FM, especially in the short-term [35]. The findings of our study second the importance of personalised care approaches [36] that validate personal experiences, provide emotional support, and actively involve patients.

### Strengths and limitations

At the core of the study was the emphasis on individual perspectives of FM, encouraging participants to articulate their lived experiences with care and everyday life. By prioritising these first-hand accounts, the study provides insights into the needs and expectations of individuals with fibromyalgia. The choice of focus groups rather than interviews was also a strength, as it fostered in-depth exchanges between participants, enriching the data through dynamic discussions. The pre-existing familiarity among participants further facilitated open dialogue. Several limitations are noted as well. First, the quality of interaction varied across the focus groups. Specifically, FG1 and FG2 exhibited more engaged and fluid exchanges compared to FG3 and FG4, potentially due to a higher number of participants in the former. This gave rise to more data from these groups and thus led to preponderance of quotes in the results from them. It should be noted that the themes emerged across all four groups, but more nuanced quotes were in the first two groups, potentially limiting the depth of conversation in those groups. This may have been due to participants’ varying time elapsed since their BRC participation, as some had completed the course over half a year earlier. Another potential contributing factor to this variation is the mode of participation in their respective BRCs: FG2 attended their BRC in person, whereas FG1, FG3 and FG4 engaged in an online format. The online group predominance was a direct result of the distancing requirement dictated by the Covid pandemic. The virtual setting may have constrained spontaneous discussion and interpersonal engagement, in turn potentially limiting the richness of data collected from these groups. Additionally, while the focus group format encouraged discussion, it may have also introduced social desirability bias, where participants adjusted their responses based on perceived group norms rather than expressing entirely individual viewpoints and thus limiting the nuances of conversation due to the feeling of wanting to belong to the group. Lastly, the study aimed to carry out a focused investigation of the experiences and perspectives of FM patients who attended a local pain management service. This limits the generalisability of the findings to this geographical region. Because of this recruitment approach, some participant voices may have been given more echo than others. For example, people with FM that are active in their care in comparison with people who would have already disengaged with care. Another point of interest in this sample is that there was only one man among participants. This is likely explained because FM is predominately female, however lived experiences may differ on these criteria. Having fewer men in the focus groups may have limited the exploration of their lived experiences.

### Future directions

The findings from this study highlight several important future research directions. There is a continued need for improving public understanding of the impact of FM. Given the reported value of social support among individuals with FM, further attention to the potential benefits of group-based interventions is warranted. Many individuals with FM in our study highlighted the positive impact of connecting with peers. Likewise, a 2024 systematic review of psychological interventions for fibromyalgia, including acceptance and commitment therapy (ACT) delivered in group format, reported small to large effect sizes across outcomes such as pain intensity, fatigue, depression, anxiety, and quality of life [37]. This underscores the need for clinical guidelines to better reflect growing evidence supporting structured group programmes in FM.

Clinicians treating individuals with FM should acknowledge the deeply personal and often invisible nature of the condition. Building trust and validating patients’ lived experiences are essential first steps toward improving care. Participants in this study reported fragmented care, limited empathy, not being believed and a strong aversion to medication—highlighting the importance of clear, compassionate communication and collaborative decision-making. We recommend that clinicians adopt a person-centred approach that considers patients’ illness narratives, beliefs about causality, and discusses non-pharmacological strategies. Past healthcare experiences and treatment expectancies should be actively explored, as they can significantly shape patients’ responses to future interventions and may enhance positive contextual effects [38]. Integrating group-based interventions may help patients feel understood, foster peer support, and provide practical coping strategies.

Further, clinical education and training on FM should reinforce the importance of holistic treatment approaches such as BRC, considering that multidisciplinary and biopsychosocial interventions significantly improve outcomes for patients with fibromyalgia [35]. Future focus group research could investigate whether individuals with FM would find patient-centred care models preferable over current care systems. Patients’ perspectives into medication need to be further explored. We observed great reluctance in our study, which is in contrast to other research, suggesting there is a complex understanding of pharmacological treatments in this patient group. An investigation into what affects medication preferences would ultimately aid patient cooperation with suggested care. Finally, individuals’ own expertise and knowledge around FM should be used in future studies by including patient representatives in the whole research process. This approach aligns with the principles of research co-design, which emphasize equitable collaboration between researchers and people with lived experience across all stages of the research cycle—from agenda setting to dissemination [39].

## Conclusion

The study highlights the deeply personal experiences of individuals living with FM, revealing persisting challenges despite the increasing awareness of the condition. Participants described universal hardships with finding understanding from their social connections. Despite these, many have developed a belief in a higher purpose and valued opportunities to better their peer network and coping strategies.

## Supporting information

S1 File Preparation and rules for focus groups. (DOCX)

S2 File Inclusivity in global research. (DOCX)
