# Supplementary material for: Has anything changed for Fibromyalgia? Focus groups with patients on their lived experiences in England
Source: PLoS One. 2026 Feb 13;21(2):e0342065. doi: 10.1371/journal.pone.0342065 (PMC12904402; doi:10.1371/journal.pone.0342065)
Supplement: S1 File — (DOCX) [file pone.0342065.s001.docx]

**Preparation and rules**

**Identifying patients’ subjective illness narratives for fibromyalgia – a focus group study**

| **PREPARATION**  **Phone call**  - parking  - significant others – happy to stay nearby to respect the privacy of others  - medication  **Room booking**  **Audio check**  **Arrival**  - Arrival time: 15 minutes before start  - we will pick them up  **Room**  - biscuits, tee  - name labels  - informed consent; questionnaire |
| --- |

**Introduction to the researcher and the project.**

**Welcome**

- Short introduction

- Make sure that patients understand – we want to learn from them. They are the experts.

**Clarify rules**

- “I will lead the focus group today”

- “I will be the time master”

- Set an alarm clock

- “This session will be audio-taped”
